# Metagenomic next-generation sequencing in diagnosing perinephric abscess infection caused by *Trichomonas vaginalis*

**DOI:** 10.1093/pcmedi/pbae027

**Published:** 2024-10-11

**Authors:** Sifen Lu, Guiming Xie, Mei Yuan, Yongzhao Zhou, Jing Wang, Juan Jiang, Wengeng Zhang, Xiaoyan He, Bojiang Chen

**Affiliations:** Precision Medicine Key Laboratory of Sichuan Province and Precision Medicine Center, West China Hospital, Sichuan University, Chengdu 610041, China; Department of Clinical Laboratory, Eighth Affiliated Hospital of Guangxi Medical University, Guigang City People's Hospital, Guigang 537100, China; Department of Clinical Laboratory, Kunming Children's Hospital, Kunming 650000, China; Department of Integrated Care Management Center, West China Hospital, Sichuan University, Chengdu 610041, China; Precision Medicine Key Laboratory of Sichuan Province and Precision Medicine Center, West China Hospital, Sichuan University, Chengdu 610041, China; Precision Medicine Key Laboratory of Sichuan Province and Precision Medicine Center, West China Hospital, Sichuan University, Chengdu 610041, China; Precision Medicine Key Laboratory of Sichuan Province and Precision Medicine Center, West China Hospital, Sichuan University, Chengdu 610041, China; Center for Clinical Molecular Medicine, National Clinical Research Center for Child Health and Disorders, Ministry of Education Key Laboratory of Child Development and Disorders, China International Science and Technology Cooperation Base of Child Development and Critical Disorders, Chongqing Key Laboratory of Pediatrics, Children's Hospital of Chongqing Medical University, Chongqing 400014, China; Precision Medicine Key Laboratory of Sichuan Province and Precision Medicine Center, West China Hospital, Sichuan University, Chengdu 610041, China

Dear Editor,

*Trichomonas vaginalis* is a common human protozoan parasite. Infection with *T. vaginalis* often manifests with symptoms such as vaginitis, itching, and dysuria, and in severe cases, infertility may occur [1, 2]. However, some infected individuals may not exhibit typical symptoms. For example, *T. vaginalis* is highly prevalent in rural South Africa and often presents without symptoms [3, 4]. Moreover, *T. vaginalis* is primarily known to infect the genitourinary system, and infection beyond this system are uncommon. A previous study reported *T. vaginalis* in the routine urine test of a 5-day-old newborn [5]. A study explored the correlation between trichomoniasis and prostate and bladder diseases [6]. Another study reported that trichomonads were detected in bronchoalveolar lavage fluid [7]. However, accurately diagnosing infectious diseases caused by trichomonads remains a significant challenge in clinical practice.

With the rapid development of metagenomic next-generation sequencing (mNGS), sequencing has emerged as an efficient method for detecting pathogens [8]. Our recent successful application of mNGS in detecting *T. vaginalis* in the drainage fluid of perinephric abscess infection adds evidence for the clinical value of the technology in diagnosing trichomonads and other atypical pathogens.

**Figure 1.**
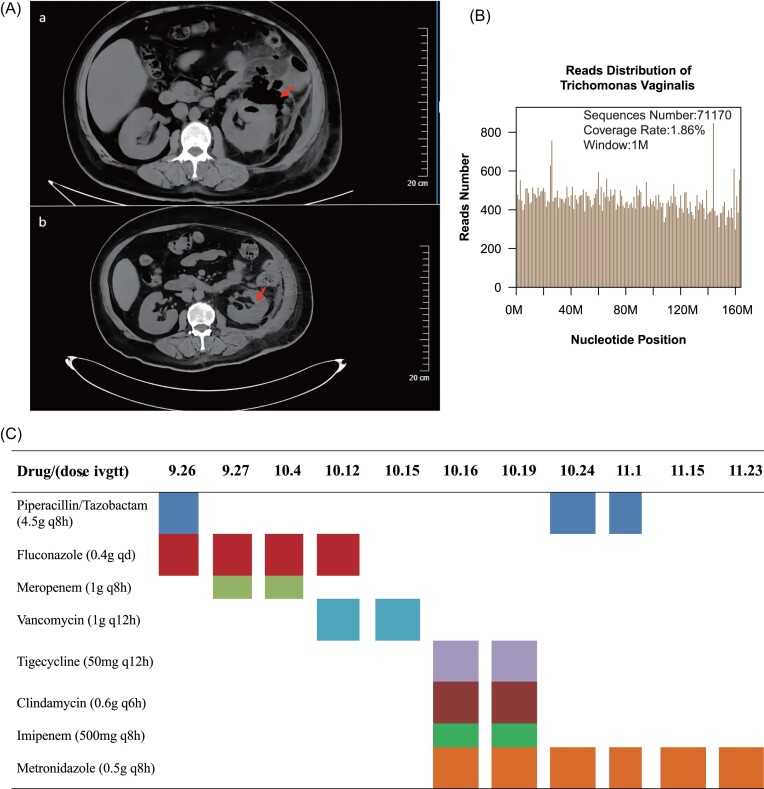
(**A**) CT scans of the abdomen upon admission showed a perirenal abscess around the left kidney, and an intermuscular abscess in the left psoas major, accompanied by multiple abscess formations. CT scans of the abdomen after anti-infective treatment showed that the inflammatory exudation and solid lesions almost completely disappeared. (**B**) mNGS result of the detected sequences distributed along the genome of *T. vaginalis*. The coverage rate is 1.86% (3 059 358/164 079 762). (**C**) Medication used for the patient during her stay at the West China Hospital of Sichuan University. Color is used to distinguish between treatments only; 9.26 refers to 26 September 2023 and 11.23 refers to 23 November 2023. q8h refers to quaque 8h, which means taking the medication once every 8 hours; qd refers to quaque day, which means taking the medication once every day;q12h refers to quaque 12h, which means taking the medication once every 12 hours; q6h refers to quaque 6h, which means taking the medication once every 6 hours; ivgtt refers to intravenously guttae.

A 49-year-old female patient was admitted to the West China Hospital of Sichuan University with a history of persistent fever for 15 days and back pain for 12 days. Her fever was of unknown origin, with the highest temperature reaching 39.0°C. Concurrently, she reported discomfort and pain on the left side of her waist. She was diagnosed with a complex urinary tract infection and perirenal infection at another hospital. She underwent anti-infection treatment at that hospital, but the specific medications administered are unknown. Unfortunately, her fever persisted and her pain symptoms intensified, so she was subsequently transferred to the West China Hospital of Sichuan University on 26 September 2023. Upon admission examination, she was conscious but exhibited signs of acute illness. Superficial lymph nodes were not palpably enlarged throughout the body. Lung auscultation revealed clear breath sounds without any dry or moist rales. The abdomen was soft, with no hepatosplenomegaly noted below the ribs. Furthermore, tenderness and rebound pain were observed in the lower left abdomen. Also, a computed tomography (CT) scan of the abdomen revealed the presence of an abscess surrounding the left kidney and within the left lumbar area, accompanied by the formation of multiple abscesses (Fig. 1A). On the same day, a left renal abscess puncture and drainage were performed under ultrasound guidance. A small amount of brown pus was drained, and tenderness was observed around the puncture site. Urinalysis revealed elevated nucleated cells ++++/HP (HP refers to high-power field of view), red blood cells ++/HP, pus cells +/HP, a red blood cell count of 2.61 × 10^12^/l, a hemoglobin level of 73 g/l, a white blood cell count of 9.73 × 10^9^/l, and a percentage of neutrophilic segmented cells of 89.3%. Urine culture indicated the presence of *Candida glabrata*, and only *Lactobacillus johnsonii* was detected in the result of the puncture fluid culture. After admission, she received a combination of piperacillin/tazobactam (4.5g q8h) and fluconazole (0.4g qd) for anti-infection treatment. On 27 September 2023, due to the severity of her infection and the inadequate control achieved with the previous antibiotic combination, the doctor switched to meropenem (1.0g q8h) for continued anti-infection treatment; however, she continued to experience persistent fever. On 16 October 2023, owing to a sudden worsening of her condition, she was transferred to the intensive care unit and underwent another surgery for incision and drainage. mNGS testing was performed with her extracted pus. After a thorough sequence alignment, the number of detected *T. vaginalis* sequences was 71 170 and the reads distribution of these sequences along the genome of *T. vaginalis* is shown in Fig. 1B. According to the result of mNGS, she was diagnosed with sepsis, perirenal abscess, *T. vaginalis* infection, and an abscess between the muscles of the left psoas major. Her condition significantly improved after treatment with metronidazole (0.5g q8h). Her medication regimen during her stay at the West China Hospital of Sichuan University is shown in Fig. 1C. After 38 days of treatment with metronidazole injection, her flank pain disappeared and there was no obvious fever. In addition, an abdominal CT scan revealed a near-complete disappearance of the inflammatory exudates (Fig. 1A). Finally, she was discharged on 23 November 2023.

*Trichomonas vaginalis* typically colonizes the lower urogenital tract and infections in the upper urinary tract are extremely rare. A previous paper reported a patient with chronic vulvovaginitis caused by *T. vaginalis*, coupled with obstructive uropathy due to kidney stones. The patient developed a perinephric abscess following injuries sustained in a motorcycle accident. Without the rapid and accurate identification of mNGS, *T. vaginalis* was identified in the discharge from the abscess through smear observation and culture. Regrettably, the patient chose to leave the hospital without further treatment and clinical data for this case are lacking [9]. The case described herein is the first case showing perinephric abscess infection caused by *T. vaginalis* using mNGS. In this case, we reported a case in which mNGS helped clinicians to accurately identify *T. vaginalis* in a patient with perirenal abscesses infection. The patient was admitted to the West China Hospital of Sichuan University with a history of persistent fever for 15 days and back pain for 12 days at another hospital. She underwent anti-infection treatment at that hospital, but her fever persisted, and her pain symptoms intensified. Upon admission to the West China Hospital of Sichuan University, a CT scan of the abdomen revealed the presence of an abscess surrounding the left kidney and within the left lumbar area, accompanied by the formation of multiple abscesses. Urine culture indicated the presence of *C. glabrata*, and only *L. johnsonii* was detected in the result of the puncture fluid culture. After admission, she received a combination of antibacterial and antifungal treatments, but she continued to experience persistent fever. Because her condition suddenly became worse, she was transferred to the intensive care unit and mNGS testing was performed with her extracted pus. Fortunately, *T. vaginalis* was identified with very high sequences numbers. Accordingly, her condition significantly improved after treatment with metronidazole and she was discharged. It is worth emphasizing in this case that only bacterial infection and fungal infection were considered at first, and the possibility of parasitic infection was ignored. If the mNGS test was used at the beginning to cover all bacteria, fungi and parasites, and *T. vaginalis* could be detected as early as possible, the patient would suffer less and the duration of the illness would be shortened. This case highlights the importance of mNGS in early diagnosis of parasites and atypical pathogens, particularly in instances where conventional diagnostic techniques struggle to confirm the pathogens. However, there are still challenges and limitations that require further technical improvement and clinical validation. Unfortunately, although *T. vaginalis* was identified by mNGS, there are no surplus fresh samples left to observe *T. vaginalis* under the microscope because the patient has been discharged from our hospital for several months and no fresh samples can be collected.

In conclusion, *T. vaginalis* was detected in a patient with perirenal abscess infection through mNGS of the pus drainage for the first time, providing a reference for early diagnosis and treatment of these infected patients in clinical practice and providing evidence that mNGS is an efficient tool for the detection of trichomoniasis and atypical pathogens. Moreover, this paper provides a basis for future research so that we can better understand the *T. vaginalis*.
